# Drug treatment program patients' hepatitis C virus (HCV) education needs and their use of available HCV education services

**DOI:** 10.1186/1472-6963-7-39

**Published:** 2007-03-08

**Authors:** Shiela M Strauss, Janetta Astone-Twerell, Corrine E Munoz-Plaza, Don C Des Jarlais, Marya Gwadz, Holly Hagan, Andrew Osborne, Andrew Rosenblum

**Affiliations:** 1 National Development and Research Institutes, Inc., 71 West 23^rd ^Street, 8^th ^Floor, New York, NY 10010, USA; 2 Beth Israel Medical Center, First Avenue at 16^th ^Street, New York, NY 10003, USA

## Abstract

**Background:**

In spite of the disproportionate prevalence of hepatitis C virus (HCV) infection among drug users, many remain uninformed or misinformed about the virus. Drug treatment programs are important sites of opportunity for providing HCV education to their patients, and many programs do, in fact, offer this education in a variety of formats. Little is known, however, about the level of HCV knowledge among drug treatment program patients, and the extent to which they utilize their programs' HCV education services.

**Methods:**

Using data collected from patients (N = 280) in 14 U.S. drug treatment programs, we compared patients who reported that they never injected drugs (NIDUs) with past or current drug injectors (IDUs) concerning their knowledge about HCV, whether they used HCV education opportunities at their programs, and the facilitators and barriers to doing so. All of the programs were participating in a research project that was developing, implementing, and evaluating a staff training to provide HCV support to patients.

**Results:**

Although IDUs scored higher on an HCV knowledge assessment than NIDUs, there were many gaps in HCV knowledge among both groups of patients. To address these knowledge gaps, all of the programs offered at least one form of HCV education: all offered 1:1 sessions with staff, 12 of the programs offered HCV education in a group format, and 11 of the programs offered this education through pamphlets/books. Only 60% of all of the participating patients used any of their programs' HCV education services, but those who did avail themselves of these HCV education opportunities generally assessed them positively. In all, many patients were unaware that HCV education was offered at their programs through individual sessions with staff, group meetings, and books/pamphlets, (42%, 49%, and 46% of the patients, respectively), and 22% were unaware that *any *HCV education opportunities existed.

**Conclusion:**

Efforts especially need to focus on ensuring that all drug treatment program patients are made aware of and encouraged to use HCV education services at their programs.

## Background

Over four million current United States residents have had acute hepatitis C virus (HCV) infection, the most common blood borne infectious disease in the U.S. [1]. In view of the ease with which HCV is transmitted by percutaneous blood contact [2], current and former drug users account for a disproportionate number of those with HCV antibodies as a result of acute infection [1,3]. Of great concern, the majority of individuals with HCV antibodies will develop chronic HCV infection [4], resulting in serious liver disease for about 20% of the chronically infected [5].

In spite of its high prevalence among injection drug users (IDUs), many individuals who inject(ed) drugs do not know their HCV serostatus [6-9]. This often reflects the fact that HCV related symptoms may only become apparent when serious viral complications have already developed, sometimes as much as several decades after exposure to the virus [10-13]. Thus, many HCV seropositive individuals remain unaware of their infection until their health is significantly compromised, treatment options are severely restricted, and they may have unknowingly transmitted the virus to others. Importantly, although the risks of contracting HCV infection are considerably less for non-injection drug users (NIDUs) than for IDUs, HCV may also be contracted when sharing contaminated straws or pipes used for sniffing or smoking drugs [14,15]. In addition, because drug users who have not injected drugs in the past may transition to this mode of drug administration in the future, it is critical that NIDUs understand the HCV related risks involved in sharing both injection and non-injection drug use equipment.

Regrettably, because drug users' access to and use of medical care is often inadequate, sometimes as a result of the discrimination they experience from physicians and other clinicians [16-18], their exposure to accurate HCV information is generally limited. In fact, many drug users are uninformed or misinformed about the mode of transmission of HCV, the existence of pharmacological therapy for HCV infection, the risks of disease progression, ways to prevent this progression (especially abstaining from alcohol), and how to avoid contracting the virus if uninfected [7,8,19]. What information they do have is often faulty or exaggerated, sometimes downplaying the seriousness of the infection, or portraying it as a frequently fatal disease [6-8,20].

Importantly, drug treatment programs are well situated to fill an important HCV education service gap among drug users by providing HCV education to their patients. In fact, our past research indicates that most drug treatment programs do offer HCV education in one or more formats (e.g., group sessions, individual sessions, videos, books/pamphlets) [21,22]. However, this education is sometimes inadequate in a variety of ways, including the limited number of patients to whom it is offered, the infrequency with which it is provided, and the lack of comprehensiveness of the topics covered [21-24]. One promising way to address these inadequacies is to better support and enable many direct care staff to provide HCV education to patients. Most drug treatment program staff understand the unique needs of the population they serve, suggesting that they are well positioned to effectively and sensitively communicate accurate and essential information about HCV and its consequences. Many of these staff, however, have limited knowledge about the virus, and some staff lack the skills to effectively communicate with patients concerning health issues, such as HCV infection. Thus, supported by a grant from the National Institute on Drug Abuse of the U.S. National Institutes of Health, we developed and are implementing a comprehensive, 6-hour, skills-based staff training (the "STOP Hep C staff training") to address many staff's HCV education and communication needs [24,25]. Details about the training, itself, are available elsewhere [26].

The STOP Hep C staff training is intended for staff in both methadone maintenance treatment programs (MMTPs) and residential drug-free programs. These treatment modalities serve many patients at high risk for HCV infection as a result of past or current drug injection (or are at serious risk for future transitioning to injection drug use) and/or the sharing of HCV contaminated non-injection drug use equipment. In addition, our HCV survey research from 2001 to 2003 with approximately 600 MMTPs and drug-free treatment programs throughout the U.S. illuminated differences in the provision of HCV services *between *modalities. In particular, MMTPs were more likely to offer HCV education services to more patients and more comprehensively than drug-free programs [22,23,27]. There were also differences *within *these modalities with regard to both aggregate patient characteristics and the HCV services offered according to region within the U.S. For example, a smaller proportion of drug-free midwestern programs participating in our past HCV survey research had a majority of IDU patients (6% of midwestern programs vs. 17%, 19%, and 24% of southern, northeastern, and western programs, respectively). Among participating MMTPs, a smaller proportion of programs in the south educated all patients about HCV (63% of southern programs vs. 77%, 76%, and 72% of western, northeastern, and midwestern programs, respectively), while a greater proportion of midwestern MMTPs did not educate any patients about the virus (14% of midwestern programs vs. 2% of southern programs and no northeastern or western programs). Thus, because of these differences between and within modalities, and because the training's impact might vary as a result of these differences, the STOP Hep C staff training research sample includes both MMTPs and drug-free treatment programs throughout the U.S.

Among the data that are needed to evaluate the impact of the STOP Hep C staff training on patients receiving substance abuse treatment in the participating programs are those that assess (a) their pre-staff training knowledge about HCV, and (b) their use of their programs' HCV education services that were already in place before the staff training took place. This will enable a comparison of post-staff training changes, if any, in patients' HCV knowledge and HCV education services use. Understanding patients' HCV knowledge and service utilization patterns has importance beyond an assessment of the impact of the STOP Hep C staff training. Such an understanding is also needed in order to inform the substance abuse treatment field of the extent to which available HCV education services are utilized (or under-utilized) in drug treatment programs, so that appropriate steps can be taken to better ensure that this vulnerable population is educated about HCV infection. We therefore performed an analysis of data collected from a sample of patients (N = 280) receiving substance abuse treatment in 14 drug treatment programs throughout the U.S. that were participating in the STOP Hep C staff training project. Using these data, collected before the STOP Hep C staff training took place, we report the results of a comprehensive examination of (a) patients' HCV knowledge, (b) their awareness and actual use of their programs' existing HCV education services, and (c) the facilitators and barriers to this service use. Given the especially heightened risk for contracting and transmitting HCV among current and former IDUs as compared with NIDUs, IDUs may have had more past exposure to HCV information and have had greater interest in continuing to learn about the virus. Thus, there may be differences between IDUs and NIDUs in their knowledge about HCV and in their awareness and actual use of HCV education services. We therefore performed our analyses by comparing data collected from NIDUs (N = 121) and IDUs (N = 159) in the 14 participating programs.

## Methods

### Programs Participating in the STOP Hep C Staff Training Research

Patients taking part in the current research were recruited from seven methadone maintenance treatment programs (MMTPs) and seven residential drug-free treatment programs that were participating in the STOP Hep C staff training project during 2005 and 2006. To be eligible to participate in the project and receive the STOP Hep C staff training, programs needed to provide drug abuse treatment services on-site and to at least 50 percent of their patients, and services could not only be for detoxification or for other short-term treatment (i.e., less than seven days). So that the training could best serve staff in programs whose patients were at considerable risk for contracting and transmitting HCV, participating programs also needed to serve patient populations in which at least 20% were drug injectors, crack smokers, and/or intranasal cocaine users. By the end of the project's data collection in 2007, programs will have been selected to include an equal number of MMTPs and drug-free treatment programs in each of the four U.S. census regions (northeast, south, midwest, west). Priority in selecting participating programs was (and continues to be) given to those programs that took part in our past HCV survey research from 2001 to 2003, which documented the HCV services provided by drug treatment programs throughout the U.S. In several instances, we included programs in the STOP Hep C staff training research that met our inclusion criteria and were recommended to us either by HCV coordinators in individual states or by other individuals involved in supporting the implementation of HCV services in drug treatment programs.

The 14 programs that participated in the STOP Hep C staff training research from September, 2005 through December, 2006 included seven residential drug-free programs: two in New York City, two in south Florida, two in north Oregon, and one in Ohio. There were also seven participating MMTPs: two in New York City, two in Ohio, one in southern California, one in southern Washington State, and one in Maryland. Data collected in a telephone interview with each program director before the STOP Hep C staff training took place documented the HCV services already in existence and available to patients at the program, including those that addressed patients' needs for HCV education. These 14 programs all offered education about HCV in one or more forms: e.g., all offered individual sessions, generally with counselors or nurses; 12 offered group sessions to provide HCV information, often as part of a health education effort conducted at the program by medical staff, counselors, or the local Health Department; 11 offered HCV education through pamphlets or other literature; and one through videos. The STOP Hep C staff training was intended to update staff's HCV knowledge and their ability to communicate this information to patients so that the HCV education services that were offered could support patients optimally. As indicated by the program directors, all of the programs offered testing for HCV antibodies (three of them on-site at the treatment program and the rest through referral), 12 offered referral for off-site treatment for HCV infection, and two offered the treatment on-site at the program.

### Participating Patients and Data Collection Procedures

At each site, the director was asked to provide a list of 20 patients who had expressed interest in taking part in the study, all of whom had an expected length of stay of at least 3 months. The length of stay requirement was instituted in order to enable a subsequent assessment of the potential impact of the STOP Hep C staff training on patients. To the extent possible, we also requested that this list of patients from each program contain an approximately equal number of HCV positive and HCV negative patients. In the residential drug-free treatment programs, the list of potential study participants typically consisted of those patients that volunteered for the study after counselors in the programs' addiction treatment groups described the research study opportunity to them. While all patients having an anticipated length of stay of 3 months or more were urged to volunteer, HCV seropositive patients were especially encouraged to do so in view of the fact that many patients in the drug-free programs were HCV seronegative (or HCV sero-unaware). In the MMTPs, most patients had an anticipated length of stay of 3 months or more and therefore satisfied our eligibility criterion. In these programs, counselors and/or nurses generally made patients aware of the research study, and HCV seronegative patients were especially encouraged to take part (as the majority of patients in the MMTPs was HCV infected).

Once compiled at the program, the list of names of potential study volunteers was provided to the study team by the program director. These patients were then scheduled to meet individually on-site at the drug treatment program with one of the well-trained research assistants from our New York based staff. This meeting enabled a more comprehensive discussion of the patients' possible participation, including the fact that patients would be compensated $15 or its equivalent in a gift card for their time. All potential study participants were given assurances regarding the voluntary nature of the research and the confidentiality of responses including the use of code numbers, rather than names, to identify study participants. Once the study was described and all questions were answered, those patients that agreed to take part in the research signed an informed consent. In fact, none of the potential participants declined to agree to participate in the interview after discussing the project with one of our project staff. In the end, 20 patients in 10 of the 14 programs took part in the research. In each of two additional programs, one of the 20 patients on the list of potential study participants was unable to take part in the research (yielding a sample of 19 from those programs). In each of the two remaining programs, 21 patients appeared on the list of potential participants supplied by the program director, and all of these patients were invited to take part in the research (yielding two program samples of 21 participants). Thus, between 19 and 21 patients at each program actually took part in the research. Data used in the analyses conducted for this paper involve those collected from patients before the staff training took place. Participating programs understood that the goal of the research was to examine the impact of an HCV staff training on patients, staff and the organization. Thus, in view of this stated goal, it is unlikely that staff would have deliberately educated participating patients about HCV or urged them to use HCV services between the time these patients volunteered for, and actually took part in the research.

Patients' participation involved the completion of a 30-minute Audio Computer Assisted Self Interview (ACASI) instrument. Using headphones, they listened as the survey questions that appeared on a computer monitor were read to them, and patients responded to these questions using a touch screen monitor. Before responding to the survey, one of the project's research assistants worked with the patients individually using a variety of practice questions, and helped them become familiar with the use of the ACASI and the instrument itself. Once the patient was comfortable with the ACASI procedure, the research assistant remained nearby to answer any questions or deal with any problems in completing the instrument. Care was taken to avoid observing patients' responses to the survey questions. The study protocol and study instruments were approved by the Institutional Review Board of the National Development and Research Institutes, Inc.

### Data Collected from Patients

Patients were asked to respond to questions involving their socio-demographics (e.g., sex, age, race, ethnicity, marital status, education level), drug use and drug treatment program history, current health (including HIV and HCV status), and health insurance coverage. They were also asked to respond to questions concerning their needs in terms of HCV education, testing, and medical care and support services, the availability of HCV services in these areas at the drug treatment program that they were currently attending, and their use of these services. In addition, they were asked to indicate, on a scale from 1 (do not agree at all) to 10 (completely agree), the extent to which they agreed with statements concerning some reasons why the use of these services may have been facilitated or made more difficult.

Patients were also asked to respond to a 20 item HCV Knowledge Assessment. As we describe in more detail elsewhere [28], the research team created this assessment to specifically address HCV-related information that is especially relevant for drug treatment programs and their patients. For each item, respondents indicated if the item was true or false or if they did not know. The 20 items include those concerning HCV transmission risk and risk behaviors; HCV diagnosis and disease progression; current HCV treatment options (including for drug dependency patients in MMTPs and other treatment programs); treatment outcomes and health maintenance if HCV infected; differences between the various types of hepatitis and the availability of vaccines for them; and HIV and HCV co-infection. A total score was obtained for each respondent on the HCV Knowledge Assessment by determining the number of items that was correctly endorsed. Each individual's score could therefore range between 0 and 20.

### Statistical Analysis

Analyses that compare the 121 NIDUs with the 159 IDUs in the 14 participating programs use chi-square tests (for categorical variables) and t-tests (for continuous variables) to test for statistical significance. P-values for results significant at the p = .05 level or less are reported.

## Results

### Characteristics of the Participating Patients

As can be seen in Table 1, close to half (45.7%) of the participating patients were female. Relative to the NIDUs, the IDUs included a significantly smaller proportion of Hispanic patients (12.6% vs. 25.6%; p = .005), and a significantly smaller proportion of non-White patients (18.9% vs. 44.6%; respectively, were Black, and 15.1% vs. 19.0%, respectively, were of races other than Black or White; p < .001). Overall, participating NIDUs were significantly younger than participating IDUs (37.7 years vs. 41.2 years; p = .005), and were less likely to be married or in a common law relationship (12.4% vs. 27.0%; p = .003). About two thirds of the sample (69.3%) had at least a high school degree or its equivalent.

**Table 1:** Characteristics of Participating Patients: Non-Injectors and Injectors

	Non-Injectors (N = 121)	Injectors (N = 159)	All Patients (N = 280)
Female (%)	43.8	47.2	45.7
Hispanic (%)**	25.6	12.6	18.2
Race (%)***			
*Black*	44.6	18.9	30.0
*White*	36.4	66.0	53.2
*Other*	19.0	15.1	16.8
Age (mean, s.d.)**	37.7 (10.3)	41.2 (10.2)	39.7 (10.4)
High School degree or above (%)	66.1	71.7	69.3
Married/Common Law (%)**	12.4	27.0	20.7
Most frequently used drugs in the 6 months before treatment (%)			
Opiates***	36.8	80.9	62.0
*Cocaine or crack*	60.7	58.6	59.5
*Alcohol*	53.0	42.7	47.1
Primary drug used in the 6 months before treatment (%) ***			
*Opiates*	33.3	70.1	54.4
*Cocaine or crack*	35.9	8.3	20.1
*Alcohol*	16.2	6.4	10.6
*Other*	14.5	15.3	15.0
Years since first used primary drug (mean, s.d.)**	17.5 (10.6)	21.2 (11.2)	19.6 (11.0)
Used primary drug every day in 6 mos. before treatment (%)***	70.1	86.6	79.6
In drug treatment before this time (%)***	52.1	73.6	64.3
- *Of these, times in drug tx. in the past (mean, s.d.)*	3.3 (2.9)	4.3 (9.4)	4.0 (7.7)
Self reported health is poor or fair (%)***	23.3	45.3	35.8
Reports having tested HIV+ (%)	7.4	5.0	6.1
Reports having tested HCV+ (rather than HCV- or HCV unaware) (%)***	6.6	56.6	35.0
Does not have health insurance (%)	47.5	36.1	41.0

** p < .01

*** p < .001

In terms of the specific drugs used in the 6 months before treatment, three out of five (59.5%) of the participating patients used cocaine or crack, and about half (47.1%) used alcohol during these 6 months. IDUs were significantly more likely than NIDUs to use opiates (80.9% vs. 36.8; p < .001) during this time. In fact, most (70.1%) IDUs identified opiates as their primary drug in the 6 months before entering drug treatment, while NIDUs were most likely to identify cocaine or crack (35.9%), opiates (33.3%), or alcohol (16.2%) [p < .001]. Overall, IDUs were significantly more likely than NIDUs to use their primary drug every day in the 6 months before entering the drug treatment program (86.6% vs. 70.1%; p = .001), and to have used their primary drug for a longer period of time (21.2 years vs. 17.5 years, p = .006). With regard to their drug injection history and practices, IDUs had been injecting for 17.8 years, on average (s.d. 12.0), and 88.1% injected drugs in the 6 months before entering the drug treatment program (data not shown in Table 1). Of this latter group of participants, two thirds (67.3%) used others' syringes and/or drug paraphernalia (e.g., cottons, rinse water) during this time, placing themselves and others at considerable risk for contracting or transmitting HCV and other blood borne infections.

Given their extensive drug use history, it is not surprising that most IDUs and NIDUs had been in a drug treatment program in the past, although this was significantly more likely to have been the case among IDUs than NIDUs (73.6% vs. 52.1%; p < .001). All participating patients that had a drug treatment program history had generally been in such programs about 4 prior times.

Overall, IDUs were significantly more likely than NIDUs to report their health as being poor or fair (45.3% vs. 23.3%; p < .001). In all, 6.1% of the patients report having tested positive for HIV, and 35.8% report having tested HCV positive, although IDUs were significantly more likely to indicate a positive test for HCV antibodies than NIDUs (56.6% vs. 6.6%; p < .001). Unfortunately, although many patients have serious health issues such as these, about two in five (41.0%) report that they do not have health insurance.

### HCV Knowledge Assessment

The 280 participating patients scored, on average, 11.1 (s.d. = 4.1) correct out of 20 on the HCV Knowledge Assessment, demonstrating limited knowledge about HCV. While IDUs scored significantly higher, on average, than did NIDUs (11.9 [s.d. = 3.8] vs. 10.1 [s.d. = 4.3]; p < .001), their scores also suggest many gaps in their knowledge about HCV.

Of note, although a significantly greater proportion of IDUs as compared with NIDUs had been in a drug treatment program before this time (suggesting that a greater proportion of participating IDUs may have been exposed to HCV education in the drug treatment program venue), there was no significant relationship between having a history of past drug treatment and a higher score on the HCV Knowledge Assessment (Pearson r = .08; p = .17). Nor was there a significant correlation for those who had such a history between their HCV Knowledge Assessment scores and the number of times they had been in a drug treatment program in the past (Pearson r = .02; p = .75).

As can be seen in Table 2, when the individual items on the HCV Knowledge Assessment are examined separately, there were statistically significant differences between the NIDUs and the IDUs on 10 of the 20 items. On almost all of these items, IDUs were significantly more likely than NIDUs to correctly endorse the items. These items had to do with HCV transmission; the duration of HCV treatment; distinguishing the various types of hepatitis infections, methadone and its interaction with HCV treatment; the absence of an HCV vaccine; the possibility of spontaneous clearance of HCV infection; and the risk of contracting HCV relative to HIV.

**Table 2:** Proportion of Correct Responses to Questions about Hepatitis C: Non-Injectors and Injectors^a^

	Non-Injectors (N = 121)	Injectors (N = 159)	All Patients (N = 280)
1. You can get the hepatitis C virus by using a hepatitis C infected person's razor.*	73.6	84.3	79.6
*2. If you have hepatitis C, drinking a few glasses of wine every day won't hurt your liver*.	74.4	81.1	78.2
3. Hepatitis C can be spread when injection drug users share their rinse water.***	61.2	85.5	75.0
*4. If a person's liver function tests are normal, the person definitely doesn't have hepatitis C*.	66.1	75.5	71.4
*5. Hepatitis C infected clients should get vaccinated for hepatitis A and hepatitis B*.	68.6	71.7	70.4
6. You'll probably get hepatitis C if you eat food from a plate that wasn't cleaned well.**	57.9	73.6	66.8
*7. Most new hepatitis C infections are caused by injection drug use*.	65.3	66.7	66.1
*8. All hepatitis C infected people will get rid of the virus if they stay on hepatitis C medications for at least one year*.	59.5	67.3	63.9
*9. The only way a doctor can really know how much the hepatitis C virus has hurt a person's liver is by doing a liver biopsy*.	62.8	63.5	63.2
10. Hepatitis C is mostly spread through risky sex.***	48.8	74.2	63.2
11. When people share needles, it's easier to get HIV than hepatitis C.**	47.9	65.4	57.9
*12. You should never take medications for hepatitis C if you have HIV*.	49.6	57.2	53.9
*13. Although the risk is low, it is possible to get hepatitis C by sharing pipes when smoking drugs*.	54.5	52.8	53.6
14. Drug users must stop taking methadone before starting HCV medication.***	40.5	62.9	53.2
15. There is a vaccine that protects against hepatitis C.***	38.8	60.4	51.1
*16. Medication to treat hepatitis C can work in active drug users*.	45.5	39.0	41.8
17. If you take medications for hepatitis C, you need to take them for many years.**	28.9	44.0	37.5
18. Bleaching needles is a good way for injection drug users to avoid getting HCV.*	38.8	25.8	31.4
*19. Most people who have hepatitis C will get serious liver disease*.	16.5	19.5	18.2
20. Some people infected with HCV can get rid of it without needing to take any medications.*	7.4	17.6	13.2

^a^Correct answers: 1. True; 2. False; 3. True; 4. False; 5. True; 6. False; 7. True; 8. False; 9. True; 10. False; 11. False; 12. False; 13. True; 14. False; 15. False; 16. True; 17. False; 18. False; 19. False; 20. True

* p < .05

** p < .01

*** p < .001

### Self-reported need for HCV education

As can be seen in Table 3, when asked to rate their need for HCV education, patients assigned a score of 5.7, on average, on a scale from 1 to 10, with '10' indicating the greatest need.

**Table 3:** Patients' Needs, Awareness and Receipt of Programs' HCV Education Services: Non-Injectors and Injectors

	Non-Injectors (N = 121)	Injectors (N = 159)	All Patients (N = 280)
Needed HCV education when entered treatment (scale from 1 to 10) (mean, s.d.)	5.4 (3.5)	6.0 (3.5)	5.7 (3.5)
Received HCV education in a group format^a ^(%)	38.7	29.6	33.6
- *Aware that HCV education is available in group sessions (%)*	62.3	54.1	57.7
- *Of those aware of its availability, received HCV education in a group format (%)*	62.1	54.8	58.3
Received HCV education in 1:1 sessions with staff (%)	26.4	33.3	30.4
- *Aware that HCV education is available in 1:1 sessions (%)*	47.1	52.8	50.4
- *Of those aware of its availability, received HCV education in 1:1 sessions (%)*	56.1	63.1	60.3
Received HCV information at the program through pamphlets/booklets^b ^(%)	34.6	41.1	37.9
- Aware that HCV information is available at the program in pamphlets/books (%)*	44.9	61.6	53.4
- *Of those aware of its availability, received HCV information in pamphlets/books (%)*	77.1	66.7	70.9
Received HCV education in some other way (e.g., videos) (%)	14.9	12.6	13.6
Received at least one form of HCV education at the program (%)	62.0	57.9	59.6
Unaware of availability of any HCV education resources at the program (%)	22.3	22.6	22.5

^a^Of the 241 patients in the 6 MMTPs and 6 drug-free treatment programs at which HCV education in a group format was offered

^b^Of the 219 patients in the 6 MMTPs and 5 drug-free treatment programs at which HCV education through pamphlets/books was offered

* p < .05

### Awareness and use of HCV education services

At 12 of the 14 programs (6 residential drug-free programs and 6 MMTPs), HCV education was offered in a group format. Among the participating patients in these programs, only three in five (57.7%) were aware that such a service was available at their programs. Of these, only 58.3% received the education in this way. Thus, only 33.6% of the patients in programs that offered HCV education in a group format received HCV education in this manner.

At each of the 14 programs, patients could receive HCV education in individual sessions with staff. In all, 50.4% of patients were aware that HCV education was available through individual sessions, but only 60.3% of the participating patients who were aware of this availability actually received HCV education in this way. Thus, only 30.4% of the patients were educated about HCV in individual sessions with staff.

Eleven of the programs (6 MMTPs and 5 residential drug-free programs) offered HCV education through pamphlets or books that the program distributed. In all, 53.4% of the patients in these 11 programs were aware that HCV education was available through books or pamphlets (although a significantly greater proportion of IDUs than NIDUs were aware of the availability of this literature: 61.6% vs. 44.9%; p = .013). As a group, only 70.9% of those who were aware of the availability of HCV information through books or pamphlets actually received HCV education in this way. Thus, only 37.9% of the patients in programs that offered HCV education through books or pamphlets were actually educated about HCV through this literature.

A small proportion (13.6%) of patients received education about HCV through videos or in other ways different from either group or individual sessions with staff, or through books and pamphlets. In all, only three in five patients (59.6%) received HCV education in *any *form, and almost a quarter (22.5%) was not even aware that any type of HCV education was available at their programs.

### Facilitators and Barriers to Using Available HCV Services

The 134 patients that received HCV education either through group sessions or individual sessions with staff, were asked to rate on a scale from 1 to 10 the extent to which they agreed with statements that described a number of possible facilitators for this receipt, with '10' indicating they completely agreed. These included "The medical staff I deal with listen to my concerns about hepatitis C" (average rating 8.1 [s.d. 2.8], median rating 9.0); "The counselors I deal with listen to my concerns about hepatitis C" (average rating 8.0 [s.d. 2.6], median rating 9.0); "The medical staff I deal with know a lot about hepatitis C" (average rating 7.6 [s.d. 3.0], median rating 9.0); "The staff I deal with make sure that I understand the information they give me about hepatitis C" (average rating 7.5 [s.d. 3.0], median rating 9.0); "The counselors I deal with know a lot about hepatitis C" (average rating 7.2 [s.d. 2.9], median rating 8.0); and "The staff I deal with bring up the topic of hepatitis C with me" (average rating 6.0 [s.d. 3.3], median rating 6.0). There were no statistically significant differences between NIDUs and IDUs concerning these facilitators.

We also asked the 92 patients that were aware of the availability of HCV education services through group and/or individual sessions with staff, but did not use at least one of these services, to rate on a scale from 1 to 10 the extent to which they agreed with statements concerning some reasons why they may not have done so. These included "If I ask about hepatitis C or go to a group about it, people might think I am infected" (average rating 3.9 [s.d. 3.5], median rating 2.0); "The person or people who provide the information know too little about hepatitis C" (average rating 3.3 [s.d. 3.1], median rating 2.0); "The person or people who provide the information use words I don't understand" (average rating 2.9 [s.d. 2.9], median rating 1.0); and "I don't want to deal with hepatitis C right now" (average rating 2.2 [s.d 2.1], median rating 1.0). In addition, the 58 patients in the 12 programs that offered HCV education in a group format who were aware of the availability of this service, but did not use it gave an average rating of 4.8 (s.d. 3.7 and median of 5.0) to "The group education about hepatitis C was given at a bad time for me." Finally, the 34 patients that were aware of the availability of literature on HCV at the program but did not use it were asked to rate, on a scale from 1 to 10, why this was the case. Respondents gave an average rating of 1.7 (s.d. 1.7 and median rating of 1.0) to "The printed information about hepatitis C is hard to understand" and an average rating of 1.4 (s.d. 1.6 and median rating of 1.0) to "The things written about hepatitis C that they give out are not in my language." There were no statistically significant differences between NIDUs and IDUs concerning any of these barriers.

## Discussion

Our results are consistent with the literature that documents drug users' limited knowledge about HCV infection, with scores on a 20-item HCV Knowledge Assessment averaging only a little better than random. Although participating IDUs averaged only 60% correct on this assessment, they scored higher than participating NIDUs overall, and a greater proportion of NIDUs correctly endorsed 9 of the 20 individual items. This may reflect IDUs' recognition of their need to be informed about HCV in view of the high prevalence of the virus among those who inject(ed) drugs, and their greater personal stake in pursuing information about HCV. The need for this information is considerable, however, for all of those at risk for contracting and transmitting the virus, including NIDUs. Participating patients in our research acknowledged this need, at least to some extent, rating it 5.7 on a scale from 1 to 10.

Given the limited knowledge about HCV on the part of these NIDUs and IDUs and their moderate recognition of their need for HCV education, it is of considerable concern that less than two thirds of them obtained such education in any form at their drug treatment programs. In particular, while all 14 of the programs offered HCV education in individual sessions with staff, 11 through books/pamphlets, and 12 offered HCV education in group sessions, actual receipt of these specific types of HCV education services ranged from only 30.4% for individual sessions with staff to 37.9% for books or pamphlets. All patients can benefit from the receipt of these services in each of these forms.

Clearly, a necessary first step in patients' use of existing HCV education services at their programs is awareness of their existence; our data show that a substantial proportion of drug treatment program patients are unaware of HCV education opportunities. Only 57.7% of the IDUs and NIDUs were aware of the availability of group sessions that covered HCV information, and only half (50.4%) was aware of the availability of individual sessions with staff to discuss HCV. This lack of awareness on the part of so many patients represents missed opportunities to address both IDUs' and NIDUs' HCV related knowledge gaps and concerns.

Importantly, of those aware of the HCV education services offered at their programs, our research did not identify many substantial barriers to obtaining this education. For some, the time at which group HCV education sessions was offered was an obstacle to attending these sessions, as was the concern that attending such a session or asking about HCV might cause others to think they had HCV infection. Overall, however, those who were educated about HCV at their programs gave high marks to counselors and medical staff in terms of listening to their concerns about HCV, knowing a lot about the virus, and making sure that patients understood the information that was given. Thus, efforts need to be directed at making certain that patients are informed about HCV education opportunities at their programs, and encouraged to make use of them.

We acknowledge a number of limitations to the research. First, the patients taking part in our research may not necessarily be representative of IDUs and NIDUs in MMTPs and residential drug-free treatment program patients nationwide, or even at their programs. With regard to the latter limitation, however, given the manner in which they were recruited, we are unaware of any specific biases in the types of patients who volunteered for the research and whose names were provided by the program directors at the participating programs. In addition, because patients were made aware of the opportunity to take part in the research by drug treatment program staff, any unknown bias that might (in fact) exist would likely be in the direction of involving more patients whose HCV knowledge and use of HCV education services was *greater *than average at each program. Thus, results from this study should be viewed as possibly *understating *the limited HCV knowledge and use of HCV education services at these programs. Second, HCV serostatus was categorized according to participants' self-report. While attempts were made to solicit volunteers for the research that included an approximately equal number of HCV seropositive and HCV seronegative patients in each of the programs, no participant was excluded from the study on the basis of her/his self-reported HCV status. Thus, combined with our emphasis on ensuring patient confidentiality, there is no specific reason to believe that the self-report is inaccurate. There is also the possibility that participants provided socially desirable responses to our questions. The use of ACASI, however, is likely to have encouraged them to respond truthfully, as their responses were not provided directly to an interviewer. Finally, with regard to the facilitators and barriers concerning the use of HCV education services, patients' responses reflect the extent to which they agreed or disagreed with statements that we provided. Thus, patients may have had other reasons that we did not ask about concerning why they did or did not use available HCV education services at their programs.

## Conclusion

Current and former drug users have significant health issues, and many of them lack health insurance coverage and face discrimination and stigma in the community. Although drug treatment programs offer an important venue to support drug users' HCV related needs, our study participants, drawn from 14 different drug treatment programs throughout the U.S. and receiving treatment in two different modalities, had limited knowledge about HCV. This, coupled with the overall satisfaction expressed by those who used HCV education services at their drug treatment programs, suggests that many drug treatment program patients may be unaware of much needed and valuable opportunities to learn about HCV-related issues in their programs. Our research also suggests the need for drug treatment programs to find ways to make more patients aware of available HCV education opportunities and encourage them to use these services. Without the current availability of a vaccine for HCV infection, and only limited success with HCV pharmacological treatment [29,30], it is imperative that drug treatment program patients obtain critical information to support reducing their risk for contracting HCV, limiting its spread to others, and maintaining their health to the greatest extent possible.

## Competing interests

The author(s) declare that they have no competing interests.

## Authors' contributions

All of the contributing authors conceived of and participated in the design of the training intervention project. SMS, JAT, and CMP coordinated the data collection activities and drafted the manuscript. All of the contributing authors read and approved the manuscript.

## Pre-publication history

The pre-publication history for this paper can be accessed here:


